# An Efficient Trap Passivator for Perovskite Solar Cells: Poly(propylene glycol) bis(2-aminopropyl ether)

**DOI:** 10.1007/s40820-020-00517-y

**Published:** 2020-08-29

**Authors:** Ningli Chen, Xiaohui Yi, Jing Zhuang, Yuanzhi Wei, Yanyan Zhang, Fuyi Wang, Shaokui Cao, Cheng Li, Jizheng Wang

**Affiliations:** 1 CAS Key Laboratory of Organic Solids, Beijing National Laboratory for Molecular Sciences, Institute of Chemistry Chinese Academy of Sciences, Beijing, 100190 People’s Republic of China; 2 University of Chinese Academy of Sciences, Beijing, 100190 People’s Republic of China; 3 Department of Physics, Xiamen University, Xiamen, 361005 People’s Republic of China; 4 CAS Key Laboratory of Analytical Chemistry for Living Biosystems, Beijing National Laboratory for Molecular Sciences, Institute of Chemistry Chinese Academy of Sciences, Beijing, 100190 People’s Republic of China; 5 School of Materials Science and Engineering, Zhengzhou University, Zhengzhou, 450000 People’s Republic of China

**Keywords:** Defects, Grain boundaries, Passivation, Stability, Perovskite solar cells

## Abstract

Perovskite solar cells (PSCs) are regarded as promising candidates for future renewable energy production. High-density defects in the perovskite films, however, lead to unsatisfactory device performances. Here, poly(propylene glycol) bis(2-aminopropyl ether) (PEA) additive is utilized to passivate the trap states in perovskite. The PEA molecules chemically interact with lead ions in perovskite, considerably passivate surface and bulk defects, which is in favor of charge transfer and extraction. Furthermore, the PEA additive can efficiently block moisture and oxygen to prolong the device lifetime. As a result, PEA-treated MAPbI_3_ (MA: CH_3_NH_3_) solar cells show increased power conversion efficiency (PCE) (from 17.18 to 18.87%) and good long-term stability. When PEA is introduced to (FAPbI_3_)_1-x_(MAPbBr_3_)_x_ (FA: HC(NH_2_)_2_) solar cells, the PCE is enhanced from 19.66 to 21.60%. For both perovskites, their severe device hysteresis is efficiently relieved by PEA.

**Electronic supplementary material:**

The online version of this article (10.1007/s40820-020-00517-y) contains supplementary material, which is available to authorized users.

## Highlights

- Poly(propylene glycol) bis(2-aminopropyl ether) (PEA) additive is introduced into the perovskite solar cells to passivate both surface and grain boundary defects, and hence improve the device efficiency and stability.
- MAPbI_3_ device with PEA exhibits significantly enhanced efficiency of 18.87%. (FAPbI_3_)_1-x_(MAPbBr_3_)_x_ device with PEA exhibits enhanced efficiency of 21.60%.
- The unsealed passivated device degrades only by 5% in PCE after being exposed to air (30 ± 5% relative humidity) for 30 days.

**Electronic supplementary material:**

The online version of this article (10.1007/s40820-020-00517-y) contains supplementary material, which is available to authorized users.

## Graphical abstract

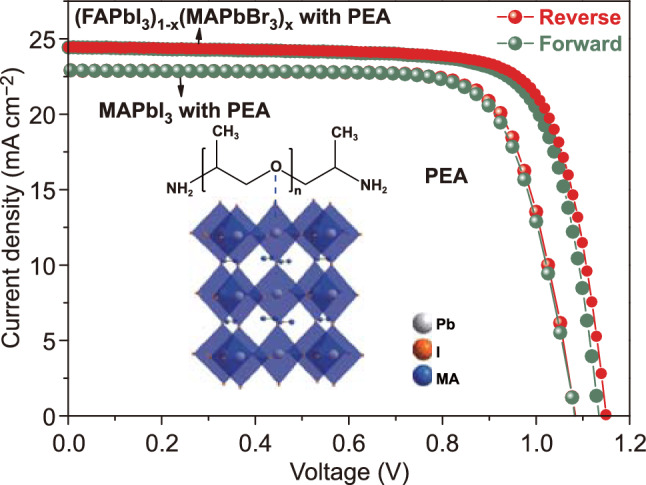

## Introduction

Perovskite solar cells (PSCs) are attractive as next-generation photovoltaic devices due to their outstanding properties such as easy preparation, low cost, and high efficiency [1–5]. PSCs are first reported in 2009 [6], and since then, rapid progress has been made in improving their performances. Recently, a certified power conversion efficiency (PCE) of exceeding 25% has been reported [7], reaching those of crystalline silicon solar cells [8]. However, a general and lasting problem for PSCs is that the perovskite films has abundant defects, at either surface or grain boundaries (GBs) [9]. These defects not only serve as recombination centers (to reduce carrier lifetime and charge extraction efficiency) [10, 11], but also facilitate the permeation of moisture and oxygen into the perovskite film, which seriously accelerates the device degradation [12–14]. Thus, defect passivation is highly desirable for achieving PSCs with both high PCE and high stability.

Surface passivation by coating molecules containing passivation groups on the surface of the perovskite layer has been proven successful for the enhancement of the device efficiency and durability. For example, the benzylamine-modified FAPbI_3_ (FA: HC(NH_2_)_2_) solar cells exhibit a champion efficiency of 19.2% and prolonged moisture durability over 2900 h [15]. By adopting a poly(methyl methacrylate) polymer layer on the MAPbI_3_ film, the device stability in moisture air is largely enhanced [16].

GB passivation via introducing additives, such as metal ions [17], small organic molecules [18, 19], polymers [20, 21], and polymer-small molecules mixtures [22], in the perovskite layer, has been a route on improving the device performance. Among these additives, polymers preferentially incorporate between GBs due to their large sizes, are able to form stable and reliable interactions with perovskite grains [23], and hence have attracted significant research interest. Zuo et al. employed poly(4-vinylpyridine) in MAPbI_3,_ improved PCEs from 16.9 ± 0.7 to 18.8 ± 0.8% and gained prolonged shelf lifetime of up to 90 days [23]. Qin et al. reported that introducing polymer PBDB-T into (CsPbI_3_)_0.04_(FAPbI_3_)_0.82_(MAPbBr_3_)_0.14_ leads to passivation of GBs. Consequently, the device shows an improved PCE of 19.85%, and is able to retain about 90% of the initial PCE after 150 days [24].

In this study, we use poly(propylene glycol) bis(2-aminopropyl ether) (PEA), which contains rich ether-oxygen groups, for the perovskite surface and GB defect passivation. The PEA molecules existing at perovskite GBs and surface chemically interact with Pb ions, retarding trap-related nonradiative recombination and enhancing charge transfer and extraction. As a result, an improvement of PCE from 17.18 to 18.87% for MAPbI_3_ solar cells is observed. Moreover, the hydrophobic PEA molecules at GBs and surface can efficiently block moisture, which significantly strengthens the device stability in air. Without any encapsulation, the MAPbI_3_ device with PEA maintains 95% of its original PCE after 30 days. The PEA-incorporated (FAPbI_3_)_1-x_(MAPbBr_3_)_x_ solar cells achieve an improved PCE from 19.66 to 21.60%. For both perovskites, PEA efficiently eliminates their original serious device hysteresis.

## Results and Discussion

### Characterization of MAPbI_3_ Films and Devices with PEA

**Fig. 1:**
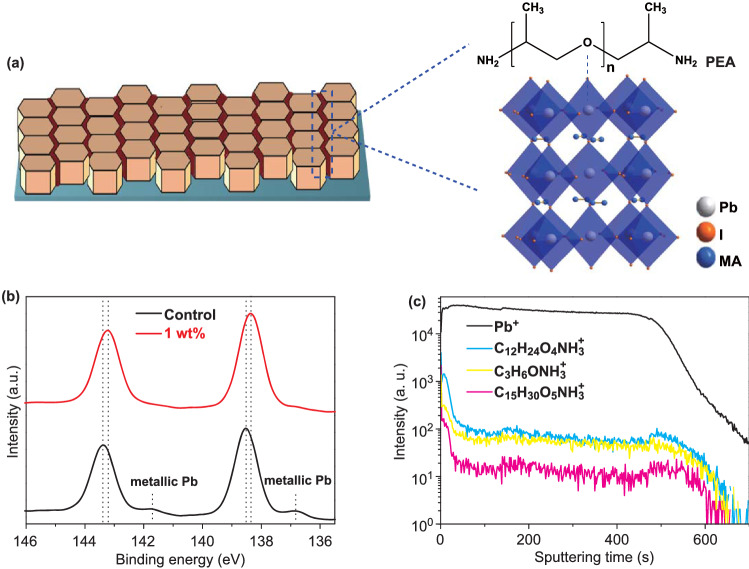
**a** Schematic diagram of the GB passivation. **b** XPS spectra of Pb 4f for the MAPbI_3_ films with and without PEA, and **c** SIMS profiles showing Pb and PEA elements from the top to the bottom of the MAPbI_3_ film with PEA

The chemical structure of a PEA molecule is shown in Fig. 1a. Pb^2+^ ions in perovskite have 6p empty electron orbits. The lone electron pairs from ether-oxygen in PEA can be delocalized to the empty orbits of Pb^2+^, forming coordination bonds [23, 25, 26]. The ether-oxygen unshared electron pair activates to form a crosslinking complex with lead ions at perovskite surface and GBs. As a result, the defect density is effectively decreased and the non-radiative recombination of the perovskite film is much inhibited [27]. This interaction is validated by Fourier transform Infrared (FTIR) spectra and X-ray photoelectron spectroscopy (XPS) measurements. Figure S1 displays the FITR spectra of PEA and the MAPbI_3_ films without (the control film) and with 1 wt% PEA. The PEA features a peak at 1110 cm^−1^, corresponding to the C–O–C stretching mode. The C–O–C peak shifts to a lower wavelength of 1064 cm^−1^ for the MAPbI_3_ film with PEA, and this is caused by the PEA molecule–perovskite interaction [28, 29]. This interaction is further verified by performing XPS measurements on the MAPbI_3_ films with and without PEA. The overview spectra are shown in Fig. S2 (the binding energy scale calibration has been done by measuring the Au 4f peak at 84.0 eV). The C 1s, N 1s, I 3d, O 1s spectra are shown in Fig. S3. The C 1s spectrum of the MAPbI_3_ film is fitted with several peaks of 285.0, 285.7, and 286.5 eV, which can be attributed to CH_3_I, surface adsorbed oxygen, and C–N bonds [19, 30]. For the C 1s spectrum of the MAPbI_3_ film with PEA, a new peak at about 287 eV appears, which can be attributed to C–O bonds in PEA [31]. And, the relative content of C–N bonds increases when PEA is presented (PEA also contains C–N bonds). The Pb 4f spectra are shown in Fig. 1b. The binding energy of Pb 4f_7/2_ (4f_5/2_) shifts from 138.52 (143.39) to 138.35 (143.21) eV. This shift confirms the decrease in cationic charge of Pb^2+^ ions in MAPbI_3_, which could be ascribed to the donation of a lone electron pair in PEA to the 6p empty orbits of Pb^2+^ [32–35]. Moreover, there are two small peaks at 136.86 and 141.75 eV in the Pb 4f spectrum of the control MAPbI_3_ film, which could be associated with the existence of metallic Pb [36]. The metallic Pb defects act as nonradiative recombination centers [37], hindering carrier transfer and collection. After PEA is added, these two small peaks diminish, implying the passivation of these defects. To study the PEA distribution throughout the MAPbI_3_ film with PEA, time-of-flight secondary-ion mass spectrometry (TOF–SIMS) tests were conducted. The “Pb^+^” from MAPbI_3_, “(C_3_H_6_O)_n_NH_3_^+^” from PEA are tracked (Fig. 1c). It is found that (C_3_H_6_O)_n_NH_3_^+^ appear at the surface of the MAPbI_3_ film and exhibit a uniform distribution in the bulk film. Furthermore, due to the large size of the polymer, there is no opportunity for the PEA molecules to incorporate into the perovskite lattice. They would mainly accumulate in the gaps between GBs [29, 38–40], where passivation occurs.

**Fig. 2:**
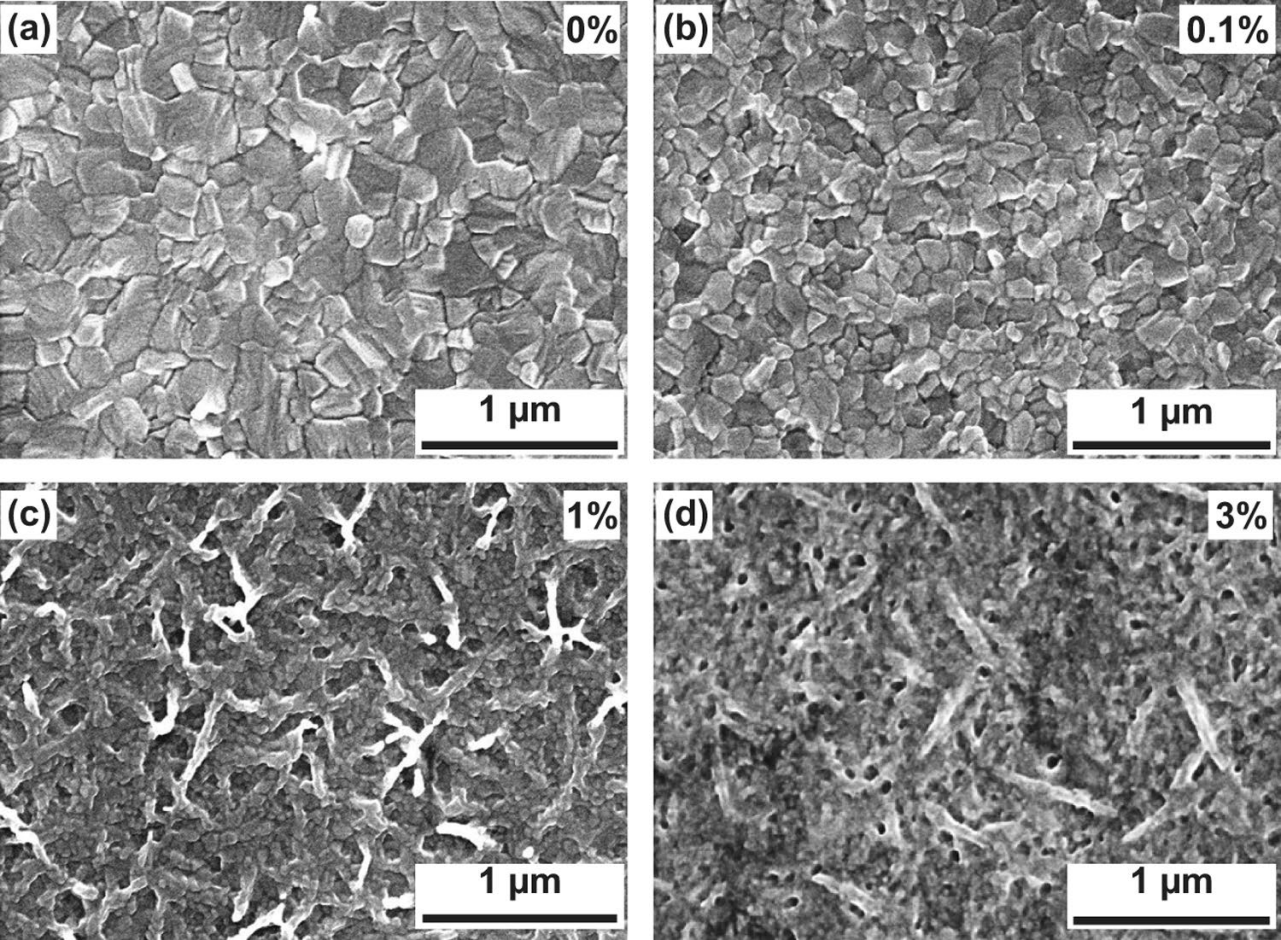
Top view SEM images of the MAPbI_3_ films with different PEA concentrations with **a** 0 wt%, **b** 0.1 wt%, **c** 1 wt%, and **d** 3 wt%

We performed top-view scanning electron microscopy (SEM) measurements on the MAPbI_3_ films with various PEA concentrations (Fig. 2a–d). The control MAPbI_3_ film presents a compact surface morphology, with grain sizes ranging from 100 to 300 nm. After 0.1 wt% PEA is added, the grain size decreases slightly. The size decrease is due to the inability of PEA to freely migrate, which has long molecule chain to hinder the growth of the perovskite crystal [40]. As the PEA concentration is increased to 1 wt%, branchlike features began to appear around GBs and on the surface, implying that the PEA molecules start to bridge the perovskite grains and occupy the perovskite surface. These branchlike features increase with increasing PEA concentration. When the PEA concentration is up to 3 wt%, most of the surface is covered by the branchlike features. Owing to the weak conductive ability of PEA, it is expected that a thick PEA layer coating on the perovskite film would weaken the electron tunneling and hinders charge transfer. In addition, we performed cross-section SEM on the MAPbI_3_ films with (1 wt%) and without PEA (Fig. S4). The control film exhibits a bumpy surface, while the MAPbI_3_ film with PEA displays a smooth surface.

**Fig. 3:**
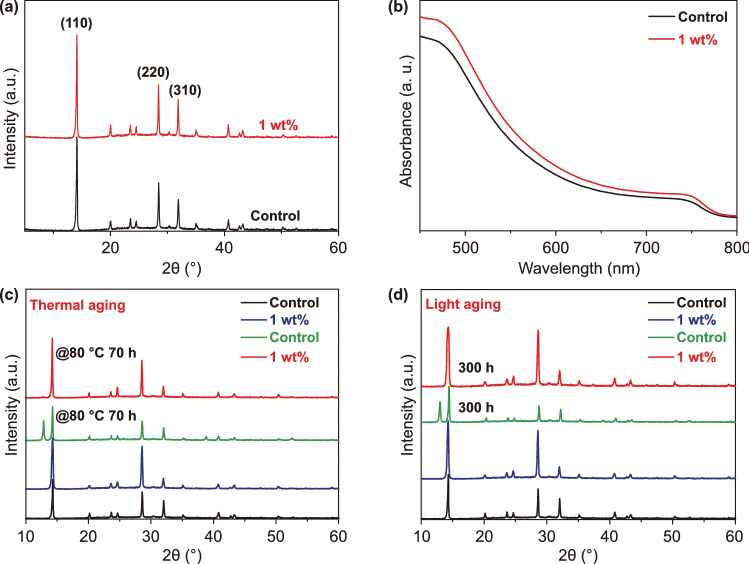
**a** XRD spectra, **b** UV–Vis absorption spectra of the MAPbI_3_ films with and without PEA. **c** XRD spectra of the MAPbI_3_ films with and without PEA, before and after thermal aging at 80 °C for 70 h, and **d** light aging for 300 h

X-ray diffraction (XRD) tests for the MAPbI_3_ films with and without PEA were conducted. Figure 3a indicates that the main diffraction peaks of the two films locate at the same positions of approximately 14.1°, 28.4°, and 31.9°, which are consistent with the diffraction peaks of (110), (220), and (310) planes of the perovskite crystal, respectively [41]. Compared to the control film, the diffraction peak intensity of the MAPbI_3_ film with PEA increases slightly, indicating an enhanced crystallinity. From the XRD spectra, we can conclude that the PEA additive is beneficial for the perovskite crystallization without affecting the perovskite crystal chemical structure. The absorption spectra of the MAPbI_3_ films with and without PEA are provided in Fig. 3b. The two films present same absorption edges, demonstrating that the band gap of the perovskite remains unchanged. The MAPbI_3_ film with PEA presents a slightly higher absorbance than the control film in the range from 400 to 750 nm, indicating a better crystallinity [42]. The thermal and light stabilities of the two films are compared (Fig. 3c, d). The control MAPbI_3_ film is unable to endure both the thermal test (80 °C for 70 h) and the light test (white light LED of 1 sun light intensity for 300 h), while the film with PEA remains undamaged. After the tests, a new XRD peak at 12.7°, which corresponds to the (001) diffraction peak of PbI_2_ [43], appears for the control film, but not for the MAPbI_3_ film with PEA. The reasons for the retarded film degradation in the presence of PEA passivation could be explained as follows: Firstly, PEA could enhance perovskite crystallization. Secondly, PEA-perovskite interaction can suppress thermally induced and light-induced ions movement and desorption from the crystal [39, 44, 45].

**Fig. 4:**
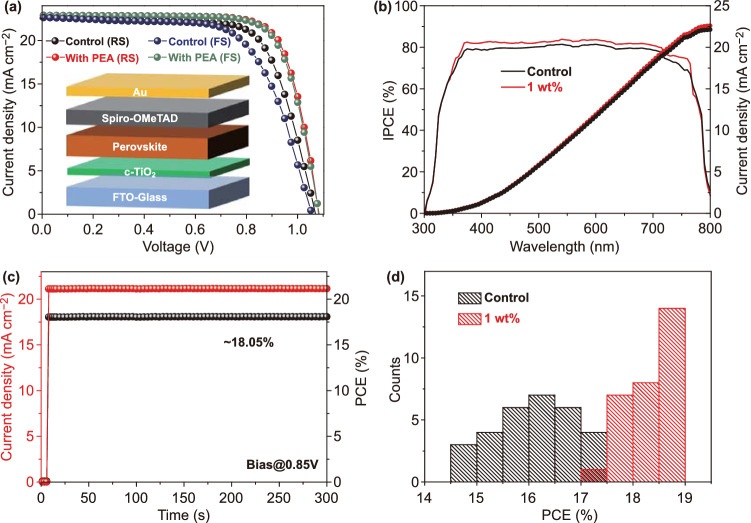
**a** *J–V* curves of the MAPbI_3_ devices with and without PEA (the inset gives the device structure). **b** IPCE spectra and integrated J_SC_ of the two devices. **c** Steady-state current density and PCE at a constant bias of 0.85 V for the device with PEA. **d** Histogram of PCE distribution

Planar heterojunction structure (PHJ) solar cells with a configuration of FTO-coated glass/TiO_2_/MAPbI_3_/Spiro-OMeTAD/Au were fabricated. By optimization, the concentration of 1 wt% PEA leads to the optimal performance of PSCs (Fig. S5 and Table S1). Figure 4a shows the best PCE from the device with 1 wt% PEA. The detailed device parameters are provided in Table S2. Under reverse scan, the MAPbI_3_ device with PEA exhibits a PCE of 18.87%, with *V*_OC_ of 1.08 V, *J*_SC_ of 22.89 mA cm^−2^, and FF of 76.3%. It outperforms the control device, which exhibits a PCE of 17.18%, with *V*_OC_ of 1.08 V, *J*_SC_ of 22.63 mA cm^−2^, and FF of 70.3%. The improved PCE is mainly ascribed to the improvement of *J*_SC_ and FF, which benefited from enhanced charge transport. In addition, hysteresis index (HI) values are calculated by Eq. (1) [20]:

1 $${\text{HI}} = \frac{{{\text{PCE}}_{{{\text{reverse}}}} - {\text{PCE}}_{{{\text{forward}}}} }}{{{\text{PCE}}_{{{\text{reverse}}}} }}$$

The device with PEA has a HI value of 0.011, which is significantly less than that of the control device (0.091). The negligible hysteresis can be attributed to efficient defect passivation by PEA. The incident photon to current conversion efficiency (IPCE) and integrated *J*_SC_ of the two devices are shown in Fig. 4b. Compared to the control device, the device with PEA has a higher spectral response in the range of 400–750 nm. The calculated integrated *J*_SC_ from the IPCE spectrum of the device with PEA is 22.58 mA cm^−2^, and that of the control device is 22.11 mA cm^−2^. Both these results concurred with those from *J–V* curves. We also recorded the photocurrent density and the PCE of the device with PEA and the control device as a function of time biased at its maximum power point voltage for 300 s. As shown in Figs. S6 and Fig. 4c, the PCEs quickly stabilize at 18.05% and 16.22%, respectively. To evaluate the reproducibility of the devices, two batches of 30 cells are fabricated. The histogram in Fig. 4d shows that the devices with PEA exhibit enhanced PCEs with narrower distributions.

**Fig. 5:**
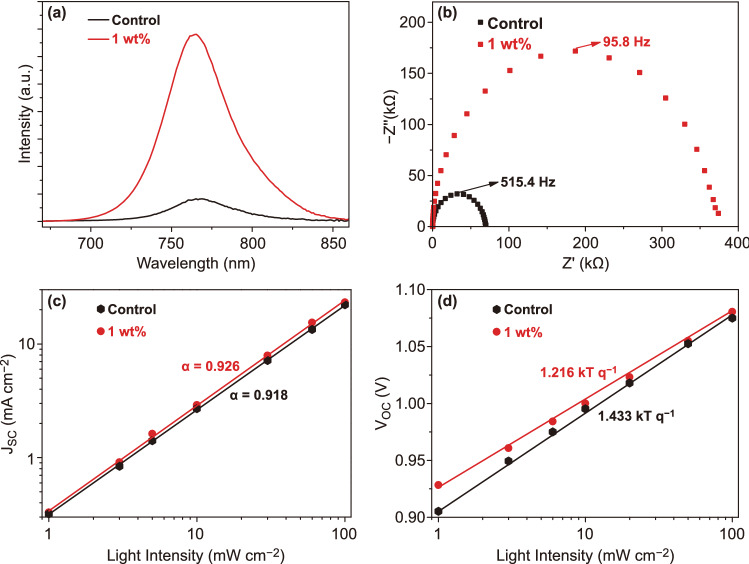
**a** PL of the MAPbI_3_ films with and without PEA on glass substrates. **b** EIS spectra, **c** *J*_SC_ versus light intensity, and **d** *V*_OC_ versus light intensity of MAPbI_3_ devices with and without PEA

Figure 5a presents the steady-state photoluminescence (PL) spectra of the MAPbI_3_ films with and without PEA on glass substrates. It is apparent that the PL peaks of the two films locate at the same energy position, and the MAPbI_3_ film with PEA shows stronger PL intensity. This demonstrates that nonradiative recombination in the perovskite film is suppressed. To explore the impact of PEA on charge carrier dynamics, time-resolved PL (TRPL) decay measurements of the two films were conducted, and the spectra are displayed in Fig. S7. The PL kinetics are fitted by the biexponential function below [Eq. (2)] [46]:

2 $$Y = A_{1} \exp \left( { - \frac{t}{{\tau_{1} }}} \right) + A_{2} \exp \left( { - \frac{t}{{\tau_{2} }}} \right) + y_{0}$$

where *τ*_1_ and *τ*_2_ are the lifetimes of the fast and slow recombination processes [47], and *A*_1_ and *A*_2_ represent the corresponding relative amplitudes. Detailed fitting parameters are summarized in Table S3. After PEA is added, τ_1_ and τ_2_ are prolonged from 4.2 and 42.5 ns to 6.0 and 69.3 ns, respectively. Furthermore, the PL lifetimes were also measured when the two films are interfaced with PCBM and Spiro-OMeTAD (Fig. S8). *τ*_1_ and *τ*_2_ are 2.8 and 6.8 ns for the PEA modified MAPbI_3_ film/PCBM, and are 3.9 and 9.4 ns for the MAPbI_3_ film/PCBM. The lifetimes are also shortened for the PEA modified MAPbI_3_ film/Spiro-OMeTAD, with *τ*_1_ and *τ*_2_ of 6.2 and 22.2 ns, as compared to 5.3 and 33.1 ns for the MAPbI_3_ film/Spiro-OMeTAD (Table S4). These results indicate suppressed charge recombination and enhanced charge transfer in the MAPbI_3_ film with PEA [48]. Electrical impedance spectroscopy (EIS) was performed on the MAPbI_3_ devices with and without PEA under open-circuit conditions in a dark environment (Fig. 5b)_._ It is apparent that the device with PEA has larger recombination resistance *R*_rec,_ indicating that the film with PEA is in favor of suppressing carrier recombination [49, 50]. In addition, the carrier lifetimes are also estimated from EIS from Eq. (3) [51–53]:

3 $$\tau = \frac{1}{2\pi f}$$

where *f* is the frequency of the highest point in EIS. The calculated *τ* is 0.31 ms for the control device, and 1.66 ms for the device with PEA. This longer carrier lifetime further demonstrates the PEA passivation benefit.

To estimate carrier trap state density in the MAPbI_3_ films with and without PEA, we performed space-charge-limited current (SCLC) measurements on hole-only and electron-only devices (Fig. S9, Supporting Information). The trap state densities are calculated from Eq. (4) [54]:

4 $$N_{t} = \frac{{2V_{{{\text{TFL}}}} \varepsilon_{0} \varepsilon_{r} }}{{qL^{2} }}$$

where *V*_TFL_ is the onset voltage of the trap-filled limit region in the dark *J–V* curves, *ε*_0_ and *ε*_*r*_ represent the vacuum permittivity and the relative dielectric constant of MAPbI_3_, respectively, and *L* is the thickness of the perovskite film. The result shows that the hole (electron) trap state density is decreased from 7.2 × 10^15^ to 4.5 × 10^15^ cm^−3^ (9.5 × 10^15^ to 5.5 × 10^15^ cm^−3^).

We then explored the variation of *J*_SC_ and *V*_OC_ under different light intensities. The power law dependence of *J*_SC_ on the incident light intensity *P*_light_ for the investigated devices is displayed in Fig. 5c, and the data are fitted by Eq. (5) [55–57]:

5 $$J_{{{\text{SC}}}} \propto P_{{{\text{light}}}}^{\alpha }$$

Without recombination, *α* value is 1. In practice, *α* will be lower than 1 because there always exists carrier recombination in a solar cell device [58–60]. The larger *α* is, the weaker the recombination is. The device with PEA has a larger *α* than the control device (0.926 vs 0.918), confirming that carrier recombination is suppressed in the device with PEA. We also investigated the dependence of *V*_OC_ on *P*_light_ and found *V*_OC_ is logarithmically dependent on *P*_light_ (Fig. 5d). The diode ideality factor n can be expressed by Eq. (6) [50, 61]:

6 $$n \approx \frac{q}{kT}\frac{{{\text{d}}V_{{{\text{OC}}}} }}{{{\text{d}}\lg \left( {P_{{{\text{light}}}} } \right)}}$$

where k, q, T are Boltzmann constant, elementary charge and temperature, respectively. For an ideal device, *n* equals 1. For a non-ideal device, *n* is larger than 1, and the larger *n*, the stronger the carrier recombination [62, 63]. The control device possesses a large *n* value of 1.433, suggesting severe carrier recombination inside the device. In contrast, the device with PEA exhibits a smaller n value of 1.216, indicating that carrier recombination is suppressed by PEA.

**Fig. 6:**
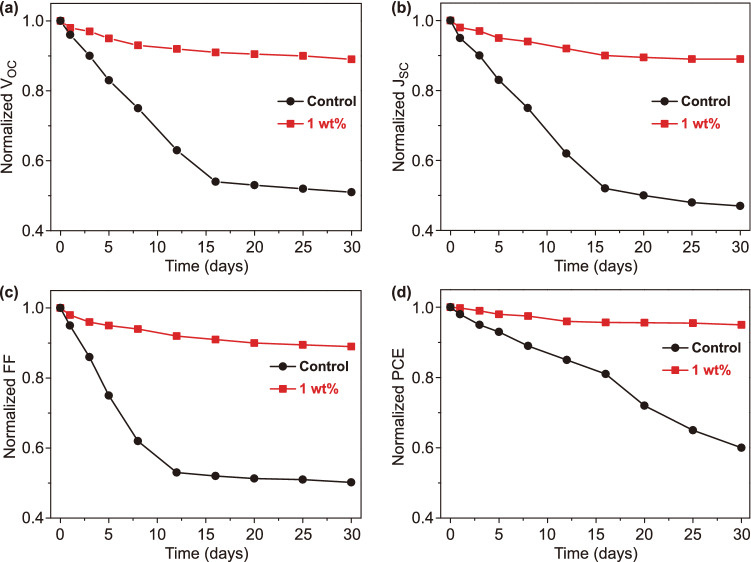
Normalized **a** *V*_OC_, **b** *J*_SC_, **c** FF, and **d** PCE of the MAPbI_3_ devices with and without PEA vs time. The devices are stored in a dark environment under ambient air with 30 ± 5% relative humidity

We then investigated the stability of the two devices in air stored in a dark environment. Figure 6a–d displays *V*_OC_, *J*_SC_, FF, and PCE variation in air (with 30 ± 5% relative humidity). The MAPbI_3_ device with PEA remains at 95% of its original PCE after 30 days, while the control device only retains 60% of its original value. This significant improvement in stability could be primarily attributed to PEA filling the GB gaps, which plays a crucial role in blocking H_2_O and O_2_. Furthermore, PEA is hydrophobic and hence would naturally repel H_2_O. The perovskite film became less hydrophilic when PEA is added (Fig. S10a, b). The MAPbI_3_ film with PEA shows a contact angle of 63.3°, while the control film shows a contact angle of 37.2°.

### Characterization of (FAPbI_3_)_1-x_(MAPbBr_3_)_x_ Devices with PEA

**Fig. 7:**
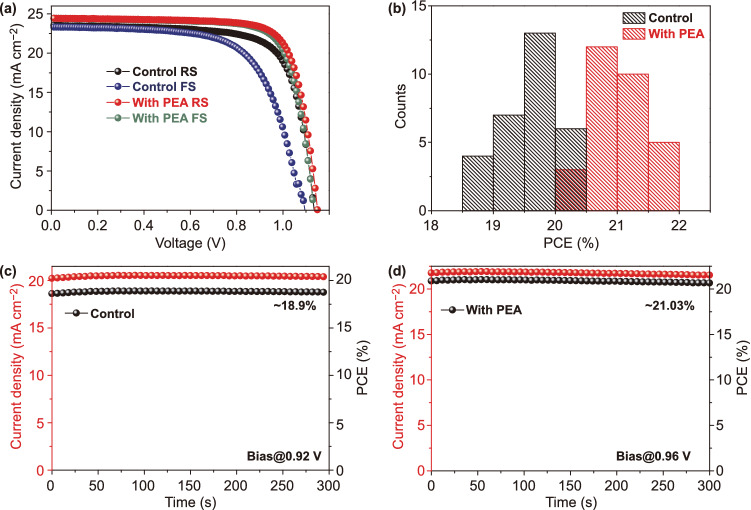
**a** *J–V* curves of the (FAPbI_3_)_1-x_(MAPbBr_3_)_x_ PSCs with and without PEA. **b** Histogram of PCE distribution. **c** Steady-state current density and efficiency at maximum power point of the device without, and **d** with PEA

Finally, we applied PEA to (FAPbI_3_)_1-x_(MAPbBr_3_)_x_ (details are described in the Experimental Section). Figure 7a demonstrates *J–V* characteristics of the corresponding devices with and without PEA. The device parameters are displayed in Table S5. The control device exhibits a PCE of 19.66%, with *V*_OC_ of 1.13 V, *J*_SC_ of 23.51 mA cm^−2^, and FF 73.7%, while the device with PEA delivers a significantly improved PCE of 21.60%, with *V*_OC_ of 1.15 V, *J*_SC_ of 24.42 mA cm^−2^, and FF of 76.9%. In addition, the control device shows a very high HI of 0.15, while the device with PEA is significantly lower, at 0.02. Their PCEs distributions are provided in Fig. 7b. Figure 7c, d shows the steady-state efficiency at maximum power point for the control device and the device with PEA, respectively. It is observed the PCE of the device with PEA stabilizes at 21.02%, while that of the control device remains at 18.90%.

## Conclusion

We employed PEA for perovskite surface and GB passivation. The ether-oxygen unshared electron pair in PEA activates to form a crosslinking complex with lead ions, resulting in the decrease in trap state density and inhibition of non-radiative recombination in the perovskite films. The MAPbI_3_-based cells with PEA obtain an improved PCE from 17.18 to 18.87%, with significantly reduced hysteresis from 9.1 to 1.1%. Moreover, the device maintains 95% of its original PCE after 30 days without any encapsulation. In contrast, the control device retains only 60% of its initial PCE under the same condition. For (FAPbI_3_)_1-x_(MAPbBr_3_)_x_-based cells, the hysteresis is significantly suppressed from 15.0 to 2.0%, and the PCE is enhanced from 19.66 to 21.60%. This work demonstrates that PEA is a very efficient trap passivator for stable high-efficiency PSCs.

## Experimental Section

### Materials

PbI_2_, MAI, FAI, MABr, MABr, Spiro-OMeTAD, and Li-TFSI are purchased from Xi’an Baolaite Technology Crop. TiCl_4_, SnO_2_ colloid precursor (15% in H_2_O colloidal dispersion), PEA, DMF, DMSO and chlorobenzene are purchased from Alfa Aesar. 4-tert-butylpyridine (TBP, 96% purity), PCBM, PEDOT: PSS are obtained from Sigma–Aldrich. All of the above materials and chemicals are used as received.

### Solution Preparation

The MAPbI_3_ precursor solution (199 mg MAI and 461 mg PbI_2_ dissolved in 800 μL DMF and 200 μL DMSO mixed solvent) is prepared in a N_2_ glove box. The PEA additive (concentration varying from 0 wt% to 3 wt%) is added in the MAPbI_3_ precursor solution and is further stirred for 2 h at 60 °C on a hotplate. The solution is filtered before use. The (FAPbI_3_)_1-x_(MAPbBr_3_)_x_ film is prepared by a two-step spin-coating process. PbI_2_ solution (760 mg PbI_2_ in 1000 μL DMF and 160 μL DMSO) is added by 0.1 wt% PEA additive and is stirred for 2 h at 60 °C. The mixed organic salt solution for the second step is 73.3 mg FAI/7.7 mg MACl/7.3 mg MABr in 1 mL IPA solvent. The Spiro-OMeTAD solution is obtained by dissolving 72.3 mg Spiro-OMeTAD, 35 μL LI-TSFI solution (260 mg mL^−1^ in acetonitrile), and 28.8 μL TBP in 1 mL of chlorobenzene.

### Fabrication of PSCs

**MAPbI**_**3**_** devices** FTO-coated glass is sequentially ultrasonically cleaned in deionized water, acetone, and isopropanol. The substrate is then dried by N_2_ flow and treated by ozone plasma for 15 min. Prior to depositing the perovskite layer, the TiO_2_ electron transport layer is fabricated by immersing the substrate in hot (70 °C) TiCl_4_ aqueous solution (with a concentration of 200 mM) for 1 h, and is further heated at 100 °C for 1 h. The MAPbI_3_ precursor is spin-coated by a successive procedure of 1000 rpm for 10 s and 4000 rpm for 30 s on top of the TiO_2_ film. In the second step, 20 s before the ending of the procedure, 100 μL chlorobenzene is rapidly dropped onto the surface of the sample. Afterwards, the sample is annealed at 100 °C for 10 min and subsequently, the Spiro-OMeTAD solution is spin-coated on the perovskite film at 4000 rpm for 30 s. The sample is then preserved in a dry dark box for 20 h. The device is completed by thermally deposition a 70-nm Au electrode.

**(FAPbI**_**3**_**)**_**1-x**_**(MAPbBr**_**3**_**)**_**x**_** devices** ITO-coated glass is sequentially ultrasonically cleaned in deionized water, acetone, isopropanol, dried by N_2_ flow, and then treated by ozone plasma for 15 min. The SnO_2_ electron transport layer is deposited on the ITO substrate by spin-coating the SnO_2_ precursor (2%, diluted by water) at 3000 rpm for 30 s, and then heated at 150 °C for 30 min. The (FAPbI_3_)_1-x_(MAPbBr_3_)_x_ film is fabricated by spin-coating the PbI_2_ solution at 1600 rpm for 10 s and then 4000 rpm for 30 s. The film is annealed at 70 °C for 2 min, followed by spin-coating the mixed solution (FAI/MACl/MABr) at 2000 rpm for 23 s and an annealing process at 140 °C for 20 min in air. The following steps for Spiro-OMeTAD film and Au electrode replicate those for the MAPbI_3_ devices.

**Hole-only devices** The PEDOT: PSS is prepared by spin-coating the solution at 2000 rpm for 50 s on the ITO substrates, and is heated at 140 °C for 10 min. The perovskite film is then deposited using the previous method. The Spiro-OMeTAD layer is then fabricated by spin-coating at 2000 rpm for 50 s. Finally, a 70-nm Au electrode underwent thermal deposition.

**Electron-only devices** The SnO_2_ layer and the perovskite layer are sequentially deposited on the ITO substrates using the previous method. The PCBM layer is then made from a precursor solution (15 mg mL^−1^ in chlorobenzene) via spin-coating at 2000 rpm for 50 s. Finally, a 100 nm Ag electrode underwent thermal deposition.

### Characterization

TOF–SIMS tests in the positive ion mode were conducted on a ToF–SIMS V instrument (ION-TOF GmbH, Münster, Germany). A pulsed 30 keV Bi_3_^+^ primary ion beam was used for the analysis with the beam current of 0.98 pA at a pulse repeating frequency of 10 kHz. The sputtering beam was 10 keV Ar_1700_^+^ with the beam current adjusted to 2 nA. The analysis area was 100 × 100 µm^2^ at the center of a crater of 250 × 250 µm^2^. During the measurements, a low-energy flood gun was used for charge compensation. FTIR spectra were tested by TENSOR-27 (Bruker, Germany), and the XPS result was obtained by ESCALAB250XI (VG, America). SEM images were obtained by S-4800(Hitachi, Japan). XRD is performed on D/max 2500 (Rigaku, Japan) with a Cu Ka radiation source (*λ* = 1.5418 Å). UV–Vis absorption spectra were characterized by UV-2600 (SHIMADZ, Japan). *J* − *V* curves were obtained by a Keithley 2400 system, with a light intensity of 100 mW cm^−2^ under an AM 1.5 solar simulator. IPCE spectra were recorded by a computer-controlled system. PL and TRPL spectra were recorded by a spectrofluorometer (FLS980, Edinburgh, England). EIS was performed on an IM6ex Electrochemical Workstation (Zahener, Germany), and the frequency range used is 1 MHz to 1 Hz.

## Electronic supplementary material

Below is the link to the electronic supplementary material.

Supplementary file1 (PDF 493 kb)
