# Supplementary material for: An Efficient Trap Passivator for Perovskite Solar Cells: Poly(propylene glycol) bis(2-aminopropyl ether)
Source: Nanomicro Lett. 2020 Aug 29;12:177. doi: 10.1007/s40820-020-00517-y (PMC7770690; doi:10.1007/s40820-020-00517-y)
Supplement: Supplementary file 1 — Supplementary file1 (PDF 493 kb) [file 40820_2020_517_MOESM1_ESM.pdf]

Supporting Information for

# An Efficient Trap Passivator for Perovskite Solar Cells: Poly (propylene glycol) bis (2-aminopropyl ether)

Ningli Chen[1, 2], Xiaohui Yi[1, 3], Jing Zhuang[1, 2], Yuanzhi Wei[1, 2], Yanyan Zhang[4], Fuyi Wang[2, 4], Shaokui Cao[5], Cheng Li[3], Jizheng Wang[1, 2, *]

[1]CAS Key Laboratory of Organic Solids, Beijing National Laboratory for Molecular Sciences, Institute of Chemistry Chinese Academy of Sciences, Beijing 100190, People's Republic of China

[2]University of Chinese Academy of Sciences, Beijing 100190, People's Republic of China

[3]Department of Physics, Xiamen University, Xiamen 361005, People's Republic of China

[4]CAS Key Laboratory of Analytical Chemistry for Living Biosystems, Beijing National Laboratory for Molecular Sciences, Institute of Chemistry Chinese Academy of Sciences, Beijing 100190, People's Republic of China

[5]School of Materials Science & Engineering, Zhengzhou University, Zhengzhou 450000, People's Republic of China

*Corresponding author. E-mail: jizheng@iccas.ac.cn (Jizheng Wang)

## Supplementary Figures

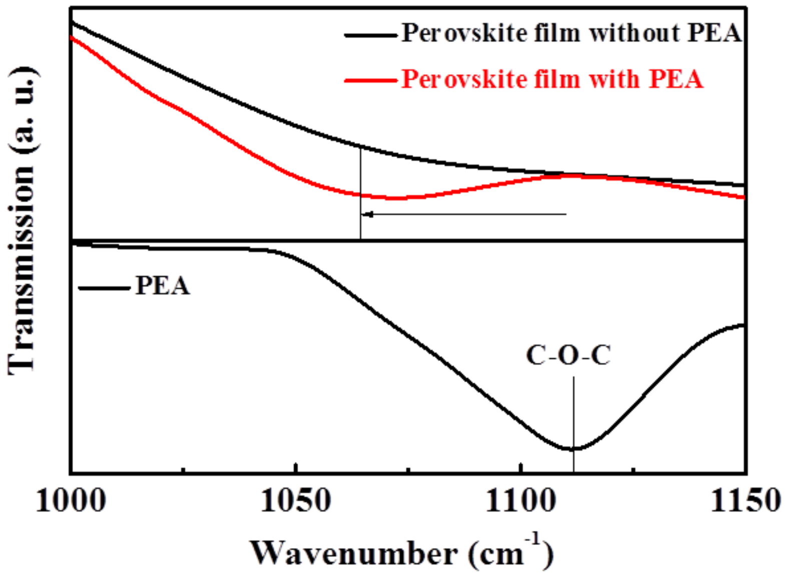


**Fig. S1** FITR spectra of PEA and the $MAPbI_3$ films with and without PEA

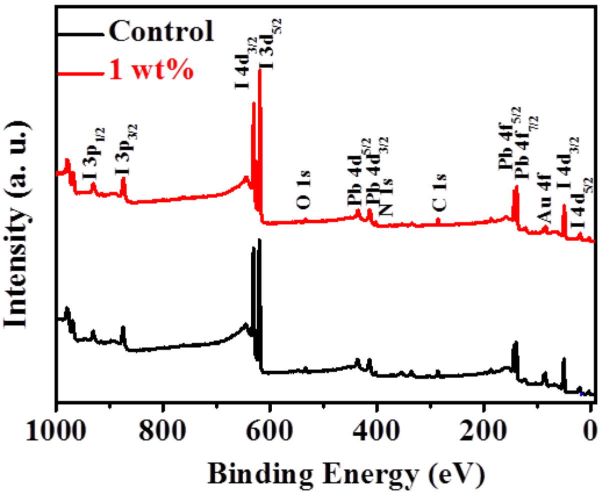


**Fig. S2** XPS of the overview spectrum for the $MAPbI_3$ films with and without PEA

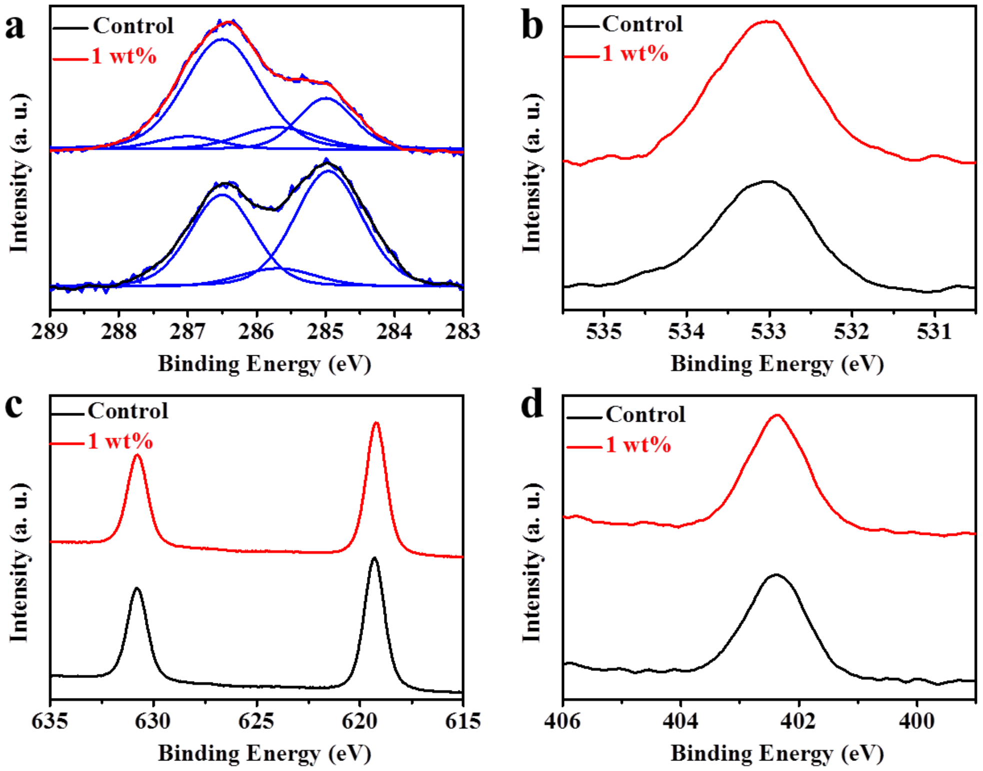


**Fig. S3** XPS spectra of (**a**) C 1s, (**b**) O 1s, (**c**) I 3d, and (**d**) N 1s for the $MAPbI_3$ films with and without PEA

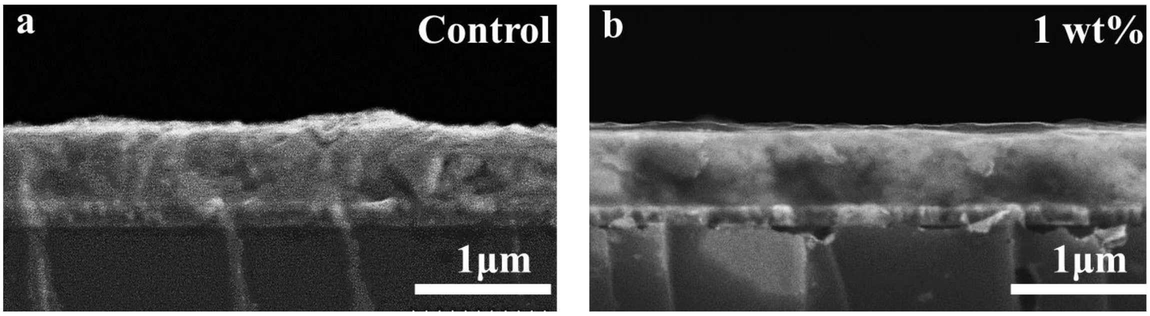


**Fig. S4** Cross-section SEM images of the $MAPbI_3$ films (**a**) without and (**b**) with PEA

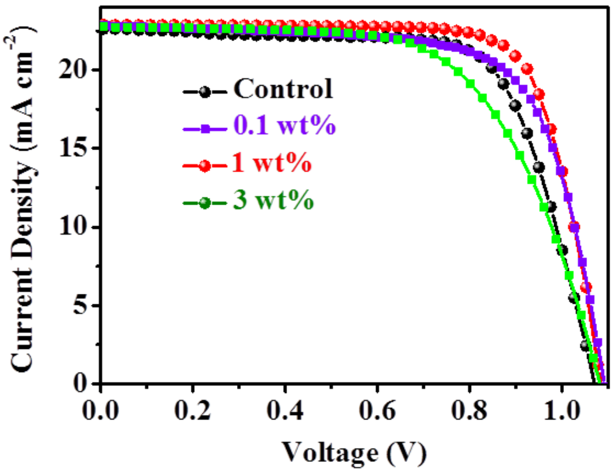


**Fig. S5** J-V curves of $MAPbI_3$ solar cells with various PEA concentration

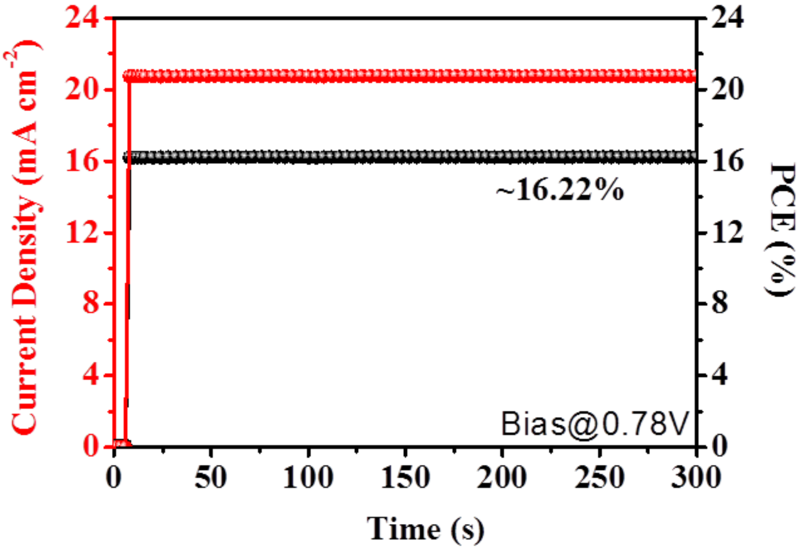


**Fig. S6** Steady-state current density and PCE for the $MAPbI_3$ device withou PEA

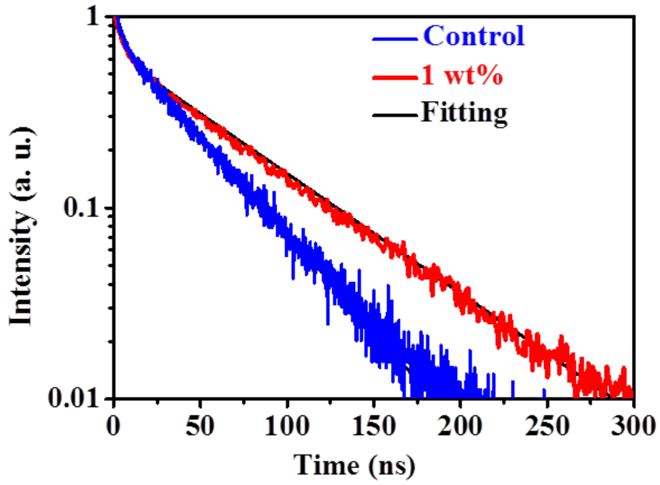


**Fig. S7** TRPL spectra of the $MAPbI_3$ films with and without PEA on glass substrates

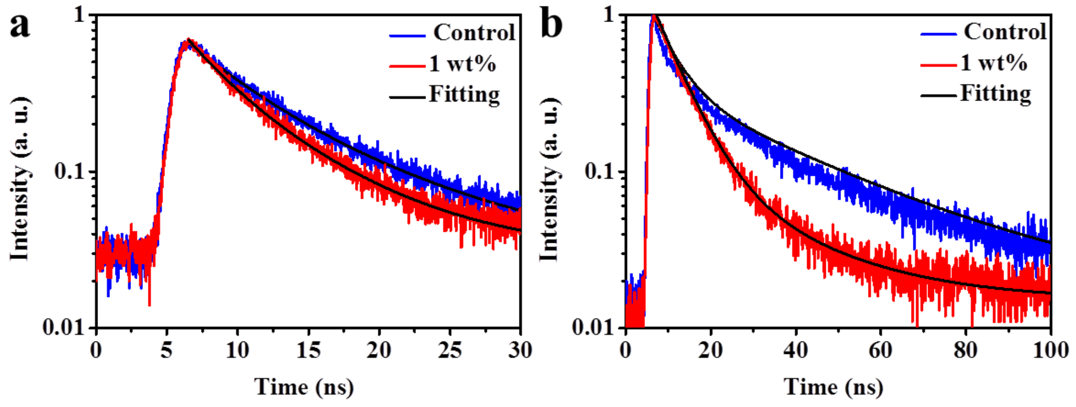


**Fig. S8** TRPL spectra of the $MAPbI_3$ films with and without PEA interfaced with (**a**) PCBM and (**b**) Spiro-OMeTAD

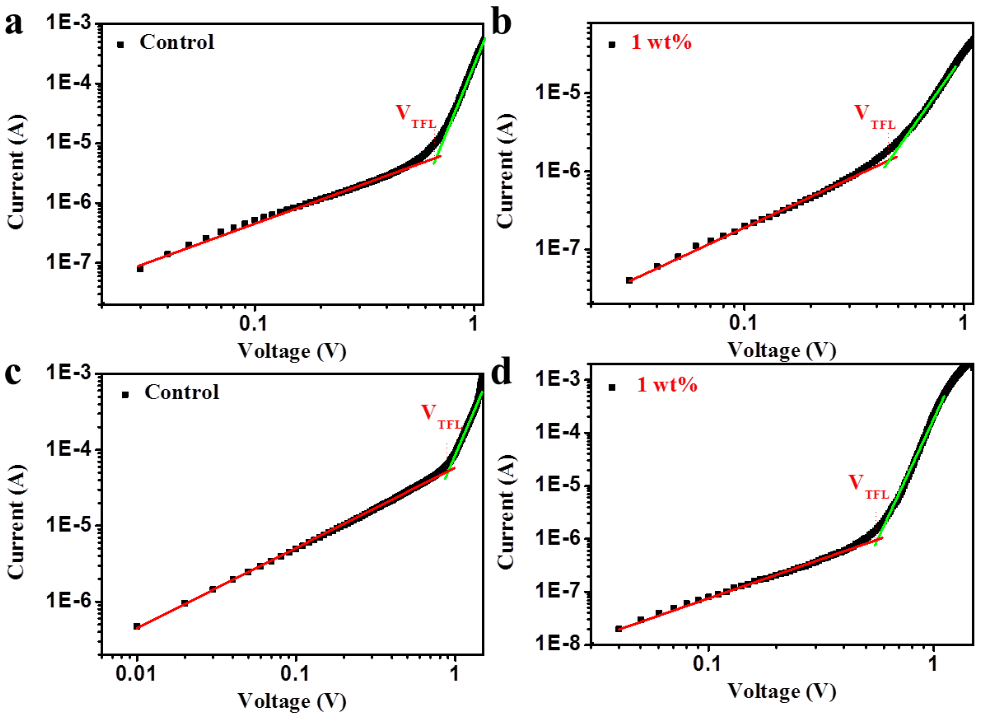


**Fig. S9** Dark Current-voltage curves for (**a, b**) hole-only devices and (**c, d**) electron-only devices

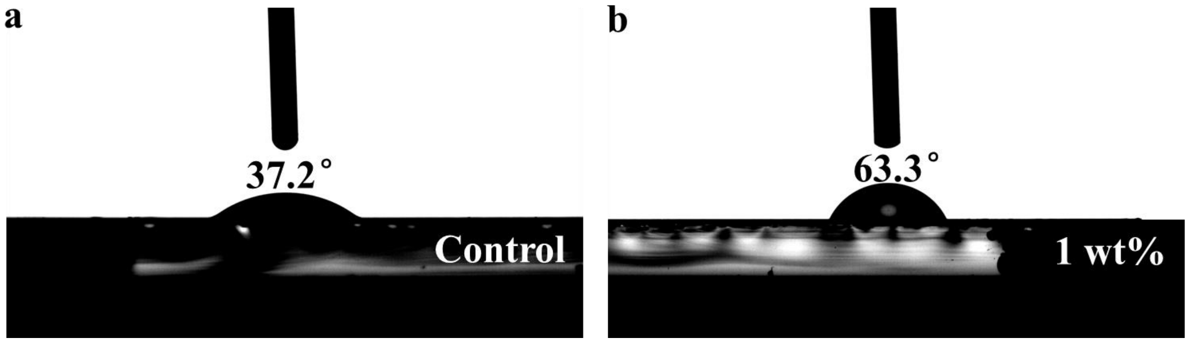


**Fig. S10** Water contact angle. (**a**) $MAPbI_3$ film without PEA. (**b**) $MAPbI_3$ film with PEA

**Table S1** Device parameters of the $MAPbI_3$ devices with various PEA concentration

| Content | $J_{SC}$ (mA cm$^{-2}$) | $V_{OC}$ (V) | FF (%) | PCE (%) |
|---|---|---|---|---|
| Control | 22.63 | 1.08 | 70.3 | 17.18 |
| 0.1 wt% | 22.82 | 1.09 | 70.3 | 17.49 |
| 1 wt% | 22.89 | 1.08 | 76.3 | 18.87 |
| 3 wt% | 22.74 | 1.08 | 62.7 | 15.41 |

**Table S2** Device parameters of the $MAPbI_3$ devices with and without PEA

| Device | $J_{SC}$ (mA cm$^{-2}$) | $V_{OC}$ (V) | FF (%) | PCE (%) | HI |
|---|---|---|---|---|---|
| Control-Reverse | 22.63 | 1.08 | 70.3 | 17.18 | 0.091 |
| Control-Forward | 22.63 | 1.05 | 65.7 | 15.61 | |
| With PEA-Reverse | 22.89 | 1.08 | 76.3 | 18.87 | 0.011 |
| With PEA-Forward | 22.89 | 1.08 | 75.5 | 18.67 | |

**Table S3** Fitting parameters for TRPL of the $MAPbI_3$ films with and without PEA

| Sample | $A_1$ | $\tau_1$ (ns) | $A_2$ | $\tau_2$ (ns) |
|---|---|---|---|---|
| Control film | 0.35 | 4.2 | 0.65 | 42.5 |
| PEA-$MAPbI_3$ film | 0.41 | 6.0 | 0.59 | 69.3 |

**Table S4** Fitting parameters for TRPL of the $MAPbI_3$ films with and without PEA interfaced with PCBM and Spiro-OMeTAD

| Sample | $A_1$ | $\tau_1$ (ns) | $A_2$ | $\tau_2$ (ns) |
|---|---|---|---|---|
| Control film/PCBM | 0.70 | 3.9 | 0.30 | 9.4 |
| PEA-$MAPbI_3$ film/PCBM | 0.78 | 2.8 | 0.22 | 6.8 |
| Control film/ Spiro-OMeTAD | 0.67 | 5.3 | 0.33 | 33.1 |
| PEA-$MAPbI_3$ film/ Spiro-OMeTAD | 0.90 | 6.2 | 0.10 | 22.2 |

**Table S5** Device parameters of the $(FAPbI_3)_{1-x}(MAPbBr_3)_x$ devices with and without PEA

| Device | $J_{SC}$ (mA cm$^{-2}$) | $V_{OC}$ (V) | FF (%) | PCE (%) | HI |
|---|---|---|---|---|---|
| Control-Reverse | 23.51 | 1.13 | 73.73 | 19.66 | 0.154 |
| Control-Forward | 23.30 | 1.09 | 65.38 | 16.64 | |
| With PEA-Reverse | 24.42 | 1.15 | 76.94 | 21.60 | 0.020 |
| With PEA-Forward | 24.40 | 1.13 | 76.49 | 21.16 | |
